# BH3 mimetic-elicited Ca^2+^ signals in pancreatic acinar cells are dependent on Bax and can be reduced by Ca^2+^-like peptides

**DOI:** 10.1038/cddis.2017.41

**Published:** 2017-03-02

**Authors:** Pawel E Ferdek, Monika A Jakubowska, Polina Nicolaou, Julia V Gerasimenko, Oleg V Gerasimenko, Ole H Petersen

**Affiliations:** 1 Medical Research Council Group, Cardiff School of Biosciences, Cardiff University, Cardiff CF10 3AX, UK; 2 Systems Immunity Research Institute, Cardiff University, Cardiff CF14 4XN, UK

## Abstract

BH3 mimetics are small-molecule inhibitors of B-cell lymphoma-2 (Bcl-2) and Bcl-xL, which disrupt the heterodimerisation of anti- and pro-apoptotic Bcl-2 family members sensitising cells to apoptotic death. These compounds have been developed as anti-cancer agents to counteract increased levels of Bcl-2 proteins often present in cancer cells. Application of a chemotherapeutic drug supported with a BH3 mimetic has the potential to overcome drug resistance in cancers overexpressing anti-apoptotic Bcl-2 proteins and thus increase the success rate of the treatment. We have previously shown that the BH3 mimetics, BH3I-2′ and HA14-1, induce Ca^2+^ release from intracellular stores followed by a sustained elevation of the cytosolic Ca^2+^ concentration. Here we demonstrate that loss of Bax, but not Bcl-2 or Bak, inhibits this sustained Ca^2+^ elevation. What is more, in the absence of Bax, thapsigargin-elicited responses were decreased; and in two-photon-permeabilised *bax*^−/−^ cells, Ca^2+^ loss from the ER was reduced compared to WT cells. The Ca^2+^-like peptides, CALP-1 and CALP-3, which activate EF hand motifs of Ca^2+^-binding proteins, significantly reduced excessive Ca^2+^ signals and necrosis caused by two BH3 mimetics: BH3I-2′ and gossypol. In the presence of CALP-1, cell death was shifted from necrotic towards apoptotic, whereas CALP-3 increased the proportion of live cells. Importantly, neither of the CALPs markedly affected physiological Ca^2+^ signals elicited by ACh, or cholecystokinin. In conclusion, the reduction in passive ER Ca^2+^ leak in *bax*^−/−^ cells as well as the fact that BH3 mimetics trigger substantial Ca^2+^ signals by liberating Bax, indicate that Bax may regulate Ca^2+^ leak channels in the ER. This study also demonstrates proof-of-principle that pre-activation of EF hand Ca^2+^-binding sites by CALPs can be used to ameliorate excessive Ca^2+^ signals caused by BH3 mimetics and shift necrotic death towards apoptosis.

Disrupted regulation of apoptosis is the hallmark of carcinogenesis allowing accumulation of further genetic mutations and acquisition of metastatic properties.^1^ Cancer cells often express increased levels of anti-apoptotic Bcl-2 (B-cell lymphoma-2) proteins, which provides additional protection against cell death signals^2, 3^ correlating with chemotherapy resistance and poor prognosis for patients.^4^

As anti-cancer therapies often target the mitochondrial apoptotic pathway regulated by the Bcl-2 family, overexpression of anti-apoptotic proteins in cancer presents one of the leading challenges to overcome for effective treatment.^5^ Mechanism of apoptosis induction in cancer is predominantly altered upstream of Bax and Bak.^6^ Therefore pharmacological suppression of anti-apoptotic Bcl-2 members leading to activation of Bax and Bak should, in principle, be capable of recovering the programmed cell death.^7, 8^

BH3 mimetics are small-molecule synthetic inhibitors of Bcl-2 and Bcl-xL, specifically developed as anti-cancer agents.^7^ They mimic activated BH3-only proteins by disrupting the heterodimerisation of anti- and pro-apoptotic Bcl-2 family members, and thus sensitising cells to apoptosis. HA14-1 was the first BH3 mimetic obtained by molecular modelling that had the ability to displace Bax from Bcl-2, followed by the induction of cell death.^7^ Later, a family of seven members of BH3 mimetics, called BH3 inhibitors (BH3Is), was developed and shown to displace Bak peptide from Bcl-xL, trigger apoptosis, cytochrome *c* release and caspase activation.^9^ A natural BH3 mimetic gossypol, isolated from the cotton plant (*Gossypium*), is also capable of inhibiting Bcl-2, Bcl-xL and Mcl-1.^10, 11^

Simultaneous application of a chemotherapeutic agent and a BH3 mimetic can potentially overcome drug resistance in cancers overexpressing anti-apoptotic Bcl-2 proteins and thus increase the treatment success rate. BH3I-2′ and HA14-1 were shown to sensitise leukaemic cells *in vitro* to TRAIL-induced apoptosis.^12^ Gossypol (AT-101) was found to increase radiation efficacy in head and neck cancer cell lines *in vitro*;^13^ and recently underwent phase II clinical trials in combination with docetaxel^14^ and with androgen deprivation therapy.^15^ A particularly interesting approach is to develop chemically tailored BH3 mimetics that interact specifically with the anti-apoptotic proteins overexpressed in a target cancer type.^8^

As exciting as the prospect of Bcl-2 inhibition might seem, accumulating evidence shows that BH3 mimetics often affect intracellular Ca^2+^ homoeostasis. Previously we have reported that HA14-1 and BH3I-2′ deplete the ER Ca^2+^ store causing a sustained elevated cytosolic Ca^2+^ concentration in pancreatic acinar cells (PACs).^16^ Others have demonstrated that HA14-1 causes Ca^2+^ deregulation in platelets, HeLa and HEK-293T cells.^17^ This immediately triggers questions about safe and specific use of BH3 mimetics, especially given the crucial role of Ca^2+^ in the regulation of a wide variety of intracellular process including muscle contraction,^18^ enzyme secretion,^19^ fertilisation,^20, 21^ cell proliferation^22^ and death.^23^ This is particularly important in the pancreas, as physiological Ca^2+^ oscillations control enzyme secretion in PACs, whereas abnormal Ca^2+^ signals are the hallmark of the initial stages of a severe necrotising disease of the pancreas – acute pancreatitis.^24, 25, 26^ This study aims to assess the effects of BH3 mimetics on Ca^2+^ handling in relation to activation of pro-apoptotic Bax in PACs. Also, we draw conclusions about the involvement of Bax in intracellular Ca^2+^ signalling and propose means to reduce the excessive Ca^2+^ signals enabling modulation of the cell death mechanisms.

## Results

### Ca^2+^ responses to BH3 mimetics in pancreatic acinar cells are dependent on Bax

**Figure 1:**
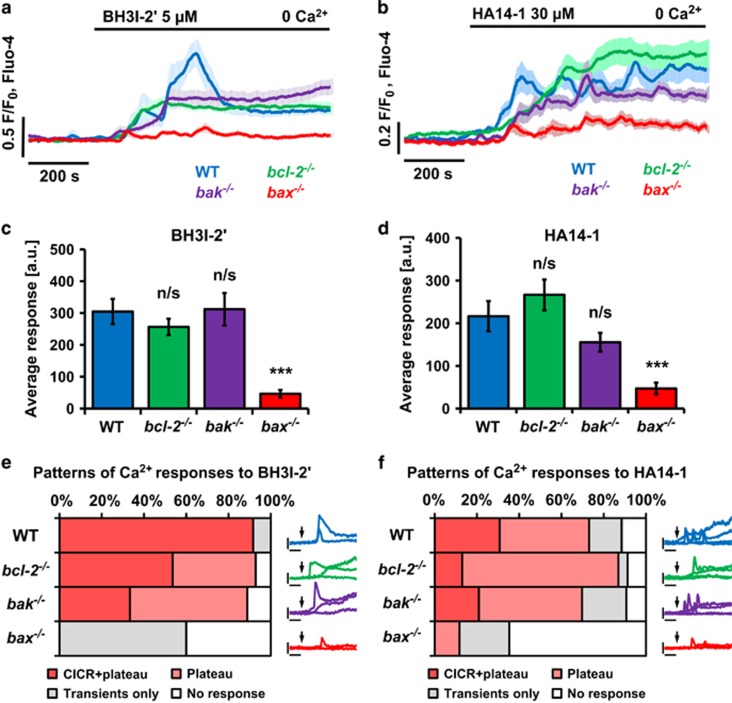
Loss of Bax markedly inhibits Ca^2+^ responses induced by BH3I-2′ and HA14-1. (**a**) Average Ca^2+^ responses (±S.E.M.) to 5 *μ*M BH3I-2′ in PACs isolated from WT mice (blue, *n*=12), *bcl-2*^−/−^ (green, *n*=28), *bak*^−/−^ (purple, *n*=27) and *bax*^−/−^ (red, *n*=20). (**b**) Average Ca^2+^ responses (±S.E.M.) to 30 *μ*M HA14-1 in PACs isolated from WT mice (blue, *n*=26), *bcl-2*^−/−^ (green, *n*=23), *bak*^−/−^ (purple, *n*=43) and *bax*^−/−^ (red, *n*=17). (**c**) The responses shown in **a** were quantitatively analysed by comparing the average Ca^2+^ areas under traces recorded between 400 and 1000 s: WT (blue, *n*=12, 304.6±39.7 a.u.), *bcl-2*^−/−^ (green, *n*=28, 256.4±25.5 a.u.), *bak*^−/−^ (purple, *n*=27, 312.0±50.9 a.u.) and *bax*^−/−^ (red, *n*=20, 46.5±11.7 a.u.). (**d**) The responses shown in **b** were quantitatively analysed by comparing the average areas under traces recorded between 400 and 1000 s: WT (blue, *n*=26, 216.5±35.1 a.u.), *bcl-2*^−/−^ (green, *n*=23, 266.6±36.1 a.u.), *bak*^−/−^ (purple, *n*=43, 155.4±22.1 a.u.) and *bax*^−/−^ (red, *n*=17, 47.0±13.6 a.u.). (**e**) Patterns of Ca^2+^ responses to BH3I-2′ averaged in **a**. Four types were identified: (1) Ca^2+^-induced Ca^2+^ release (CICR) type followed by a sustained elevation (plateau), (2) Ca^2+^ plateau with no CICR, (3) Ca^2+^ transients (CICR) with no plateau formation, (4) no response. Insets show sample traces of each identified type of response (scale: *x* axis: 200 s; *y* axis: 1.0 *F/F*_0_, Fluo-4). (**f**) Patterns of Ca^2+^ responses to HA14-1 averaged in **b**. The responses were classified as in **e**. Insets show sample traces of each identified type of response (scale: *x* axis: 200 s; *y* axis: 1.0 *F/F*_0_, Fluo-4)

**Figure 2:**
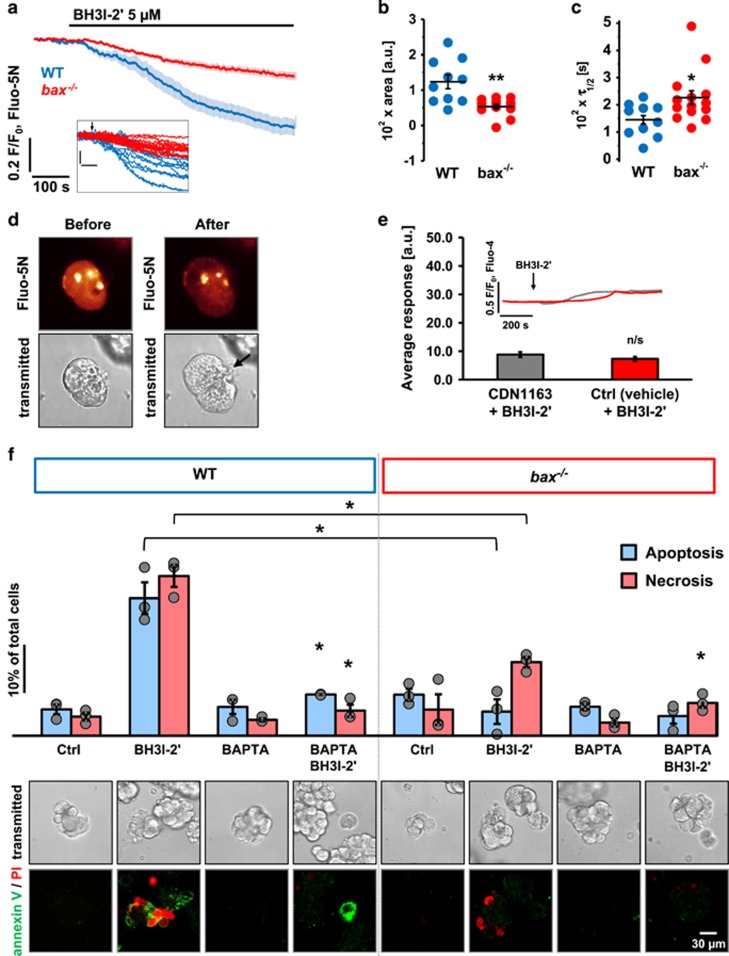
Loss of Bax reduces BH3I-2′-induced Ca^2+^ leak from the ER and cell death in PACs. (**a**) Average traces (±S.E.M.) showing Ca^2+^ release from the ER of permeabilised WT (blue, *n*=10) and *bax*^−/−^ (red, *n*=14) PACs induced by 5 *μ*M BH3I-2′. Inset shows individual traces. (**b**) Dot chart shows individual and average (±S.E.M.) response areas below the baseline calculated between 100 and 600 s for the traces from **a**: WT (123,6±19.7 a.u.) and *bax*^−/−^ (53.3±6.3 a.u.). (**c**) Dot chart shows the half-times (*τ*_1/2_) of the reduction in fluorescence of the Ca^2+^ indicator in the ER (and thus [Ca^2+^]_ER_) towards the final levels calculated for the traces depicted in **a**. (**d**) Images of a PAC doublet before and after two-photon permeabilisation. Black arrow shows the site of permeabilisation. (**e**) Bar chart shows average areas under traces (±S.E.M.) calculated between 200 and 1000 s for *bax*^−/−^ cells pre-incubated for 2 h with 10 *μ*M CDN1163 (grey, *n*=61, 88.3±9.0 a.u.) or with 0.05% DMSO (vehicle control, red, *n*=89, 73.3±7.1 a.u.) and then treated with 5 *μ*M BH3I-2′. Inset shows averaged traces (±S.E.M.). (**f**) Apoptosis and necrosis induced by 30 min incubation with 5 *μ*M BH3I-2′ in WT and *bax*^−/−^ PACs with or without 15 min pretreatment with 25 *μ*M BAPTA. Blue bars represent apoptotic cells, and red – necrotic. *N*=3 for all the groups; individual values are indicated with grey dots. Below each pair of bars, sample images show morphology (transmitted light images) and typical annexin V (green), and propidium iodide (red) staining of PACs in the experiment

BH3I-2′ and HA14-1 were shown to induce a slow Ca^2+^ release from the intracellular stores.^16^ Pharmacological inhibition of inositol 1,4,5-triphosphate receptors (IP_3_Rs) and ryanodine receptors (RyRs) led to a decreased Ca^2+^ release from the ER of permeabilised PACs but did not completely block it. This indicates that IP_3_Rs and RyRs were not the primary source of BH3 mimetic-elicited Ca^2+^ release but merely amplified it.^16^ Here PACs, isolated from wild-type (WT) mice as well as animals with a loss-of-function mutation in one of the following genes: *bcl-2*, *bak* or *bax* were treated with 5 *μ*M BH3I-2′ (Figure 1a) or 30 *μ*M HA14-1 (Figure 1b) in the absence of extracellular Ca^2+^. In WT, *bcl-2*^−/−^ and *bak*^−/−^ cells, these BH3 mimetics caused sustained elevations of the cytosolic Ca^2+^ concentration ([Ca^2+^]_i_), whereas in *bax*^−/−^ cells the Ca^2+^ signal generation was largely abolished (Figures 1a and b). Quantitative comparison of the responses to BH3I-2′ (Figure 1c) and HA14-1 (Figure 1d) confirmed that loss of Bax, but not Bcl-2 or Bak, dramatically inhibited the BH3 mimetic-induced elevation of the [Ca^2+^]_i_ (*P*<0.001). The responses to BH3I-2′ or HA14-1 in *bax*^−/−^ cells, if present at all, were mainly short-lasting Ca^2+^ transients, whereas sustained elevation was mostly inhibited (Figures 1e and f). Further, measurements of Ca^2+^ release from the ER in two-photon-permeabilised PACs revealed that BH3I-2′ not only appears to release less Ca^2+^ from the ER of *bax*^−/−^ compared to WT cells (*P*<0.01; Figures 2a and b) but also that the apparent rate of release was slower than in WT cells. Consequently, *τ*_1/2_ for *bax*^−/−^ (226.5±25.9 s) cells was higher than for WT cells (145.1±19.3 s; *P*<0.05; Figure 2c), which translates into longer time needed for the fluorescence of the Ca^2+^ indicator to decrease by half the difference between baseline and final values. Images of a PAC doublet before and after laser permeabilisation are depicted in Figure 2d. Increasing the ER store loading with CDN1163, a postulated allosteric activator of SERCA2b (sarco/endoplasmic reticulum Ca^2+^-ATPase, isoform 2b),^27^ did not significantly affect the responses to BH3I-2′ in *bax*^−/−^ cells (Figure 2e). Finally, BH3I-2′ increased apoptosis in WT cells by ~20.6±3.8% and necrosis by 26.1±2.7% over control levels; these effects were completely abolished by intracellular Ca^2+^ chelation with BAPTA (*P*<0.05; Figure 2f; bars and corresponding images). In *bax*^−/−^ cells, BH3I-2′ did not affect apoptosis and only slightly increased necrosis (by 8.8±3.8%); the latter was completely inhibited by BAPTA (*P*<0.05; Figure 2f). The effects of BH3I-2′ on cell death were substantially less pronounced in *bax*^−/−^ cells compared to WT cells (*P*<0.05 for both apoptosis and necrosis; Figure 2f).

### Loss of bax reduces Ca^2+^ release from the ER

**Figure 3:**
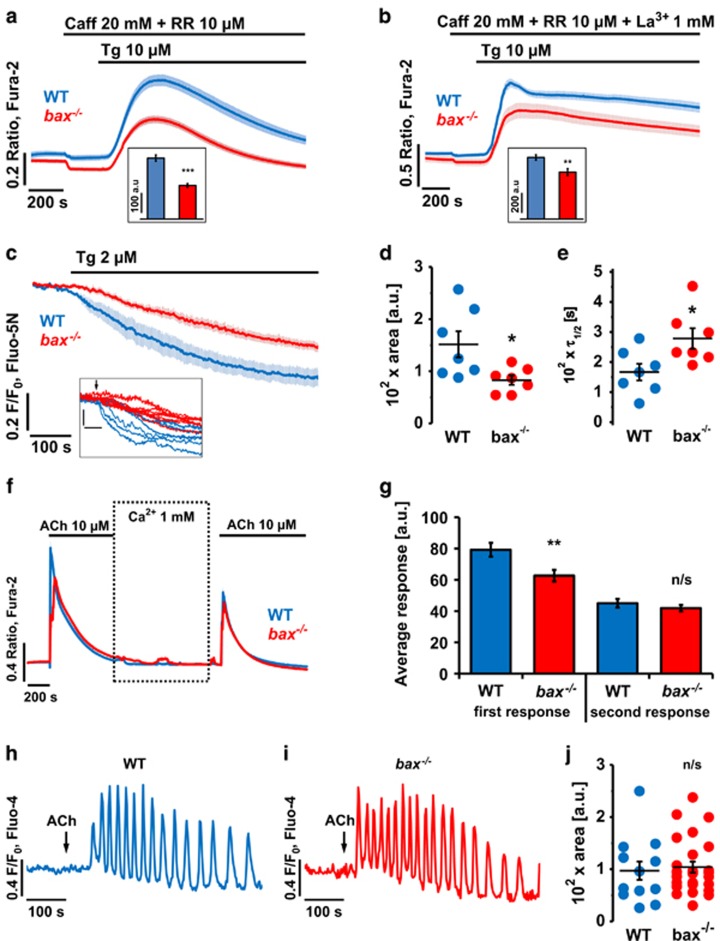
Loss of Bax reduces Ca^2+^ release from the ER. (**a**) Average Ca^2+^ responses (±S.E.M.) induced by 10 *μ*M thapsigargin (Tg) in WT (blue, *n*=9) and *bax*^−/−^ (red, *n*=12) PACs. IP_3_Rs and RyRs were inhibited by 20 mM caffeine (Caff) and 10 *μ*M ruthenium red (RR), respectively. Inset: average areas under traces calculated between 400 and 1400 s for the individual recordings in WT (305.4±15.5 a.u.) and *bax*^−/−^ (168.7±8.9 a.u) cells. (**b**) Average Ca^2+^ responses (±S.E.M.) induced by 10 *μ*M Tg in WT (blue, *n*=24) and *bax*^−/−^ (red, *n*=21) PACs in the presence of 20 mM Caff and 10 *μ*M RR under inhibition of Ca^2+^ extrusion by 1 mM La^3+^. Inset: average areas under traces (±S.E.M.) calculated between 400 and 1400 s for the individual recordings in WT (616.3±26.5 a.u.) and *bax*^−/−^ (469.4±35.9 a.u) cells. (**c**) Average traces (±S.E.M.) showing Ca^2+^ release from the ER of permeabilised WT (blue, *n*=7) and *bax*^−/−^ (red, *n*=7) PACs induced by 2 *μ*M Tg. Inset shows individual traces. (**d**) Dot chart shows individual and average (±S.E.M.) areas calculated between 100 and 600 s for the traces from **c**: WT (151.7±25.1 a.u.) and *bax*^−/−^ (83.1±9.1 a.u.). (**e**) Dot chart shows the half-times (*τ*_1/2_) of the reduction in fluorescence of the Ca^2+^ indicator in the ER (and thus [Ca^2+^]_ER_) towards the final levels calculated for the traces depicted in **c**. (**f**) Average Ca^2+^ responses (±S.E.M.) to two applications of supramaximal doses of ACh (10 *μ*M) in the absence of extracellular Ca^2+^, separated by partial reloading in 1 mM extracellular Ca^2+^ for 15 min. (**g**) Average areas under traces (±S.E.M.) calculated in the first 100 s after application of ACh for the individual recordings averaged in **f**. (**h**) 100 nM ACh induces Ca^2+^ oscillations in WT PACs (representative trace, *n*=12). (**i**) 100 nM ACh induces Ca^2+^ oscillations in *bax*^−/−^ PACs (representative trace, *n*=25). (**j**) Dot chart shows individual and average (±S.E.M.) response areas calculated between 100 and 600 s of each recording from WT and *bax*^−/−^ cells

The effects of Bax on intracellular Ca^2+^ homoeostasis were investigated in PACs that had their ER stores depleted with the SERCA blocker thapsigargin under simultaneous inhibition of IP_3_Rs (by 20 mM caffeine) and RyRs (by 10 *μ*M ruthenium red).^16, 28^ Average traces (Figure 3a) and average areas of responses (Figure 3a, inset) demonstrate that [Ca^2+^]_i_ elevation caused by the ER depletion was significantly smaller in *bax*^−/−^ cells than in WT cells (*P*<0.001). Inhibition of Ca^2+^ extrusion by 1 mM La^3+^ preserved the difference in size of the thapsigargin-induced Ca^2+^ release between WT and *bax*^−/−^ cells (*P*<0.01; Figure 3b) indicating that this difference was not due to enhanced cytosolic Ca^2+^ extrusion in *bax*^−/−^ cells. Further, in two-photon-permeabilised PACs, thapsigargin also released less Ca^2+^ from the ER of *bax*^−/−^ than from WT cells (*P*<0.05; Figures 3c and d) and the apparent rate of release was slower in *bax*^−/−^ compared to WT cells (*τ*_1/2_=278.3±34.2 s *versus* 166.6±27.7 s; *P*<0.05; Figure 3e). Emptying the ER store with a supramaximal dose of acetylcholine (ACh, 10 *μ*M) resulted in only very slightly diminished responses in *bax*^−/−^ cells in the first 100 s (62.7±3.7 a.u.) compared to WT cells (79.2±4.4 a.u.; *P*<0.01; Figures 3f and g). The second emptying of the ER store, preceded by partial reloading in 1 mM extracellular Ca^2+^, showed no statistically significant difference between WT (45.0±2.8 a.u.) and *bax*^−/−^ cells (41.9±2.0 a.u.; *P*=0.38; Figures 3f and g), indicating that ER store refilling was not substantially altered by loss of Bax. Finally, the responses to physiological doses of ACh (100 nM) were essentially unaffected as shown by sample traces (Figures 3h and i) and the response areas (Figure 3j).

### CALPs inhibit Ca^2+^ entry in pancreatic acinar cells

Calcium-like peptides (CALPs) are short peptides designed by inversion of the hydrophobic pattern of EF hand Ca^2+^-binding sites,^29^ which generates molecules of a complementary surface contour, capable of interacting with the sequences of interest.^30, 31^ Cell permeable CALP-1 and CALP-3 can functionally mimic increased [Ca^2+^]_i_ by modulating the activity of calmodulin, Ca^2+^ channels and pumps.^32^

**Figure 4:**
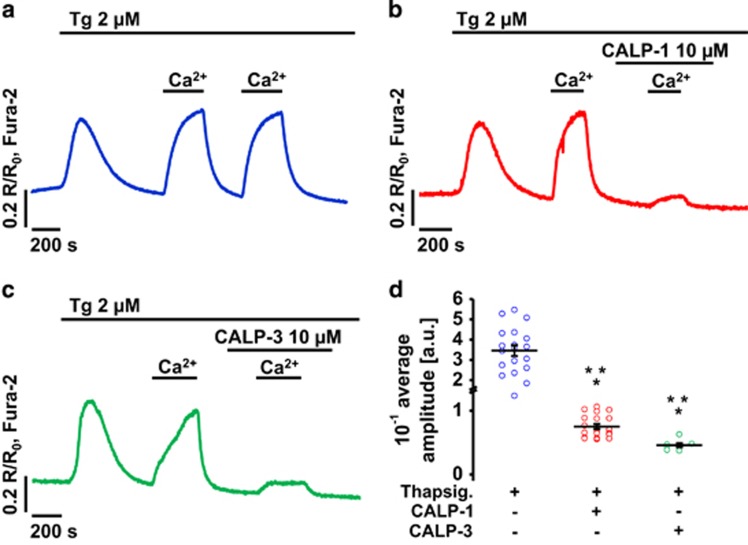
CALPs inhibit Ca^2+^ entry in PACs. (**a**) The ER store was emptied with thapsigargin (Tg) in the absence of extracellular Ca^2+^. Two consecutive applications of 10 mM extracellular Ca^2+^ triggered Ca^2+^ entry into the cytosol (sample trace, *n*=19); control experiment for **b** and **c**. (**b**) The ER store was emptied with Tg in the absence of extracellular Ca^2+^. Application of 10 mM extracellular Ca^2+^ triggered Ca^2+^ entry into the cytosol. The first response to 10 mM Ca^2+^ was an internal control; the second response was inhibited in the presence of 10 *μ*M CALP-1 (sample trace, *n*=19). (**c**) The ER store was emptied with Tg in the absence of extracellular Ca^2+^. Application of 10 mM extracellular Ca^2+^ triggered Ca^2+^ entry into the cytosol. The first response to 10 mM Ca^2+^ was an internal control; the second response was inhibited in the presence of 10 *μ*M CALP-3 (sample trace, *n*=7). (**d**) The chart shows amplitudes (individual points and mean ±S.E.M.) of the second Ca^2+^ entry response calculated in control experiments (0.346±0.027 a.u., *n*=20) and in the presence of 10 *μ*M CALP-1 (0.075±0.004 a.u., *n*=20) or 10 *μ*M CALP-3 (0.046±0.003 a.u., *n*=7)

Emptying the ER Ca^2+^ store with thapsigargin leads to opening of store-operated Ca^2+^ entry (SOCE) channels in the plasma membrane and influx of Ca^2+^ into the cytosol.^33^ As seen in Figures 4a–c, thapsigargin elicits an increase in [Ca^2+^]_i_ which, in the absence of external Ca^2+^, is transient due to the extrusion of Ca^2+^ by the plasma membrane Ca^2+^ pumps. When Ca^2+^ is added to the external solution, [Ca^2+^]_i_ increases again, due to SOCE (Figures 4a–c). In the absence of CALPs, the first and second application of 10 mM Ca^2+^ induced similar [Ca^2+^]_i_ elevations (Figure 4a). Addition of low concentrations (10 *μ*M) of CALP-1 (Figure 4b) or CALP-3 (Figure 4c) before the second application of 10 mM Ca^2+^ substantially inhibited Ca^2+^ influx, as shown by markedly reduced amplitudes (*P*<0.001; Figure 4d).

### CALPs reduce excessive cytosolic Ca^2+^ signals and necrosis elicited by BH3I-2′

**Figure 5:**
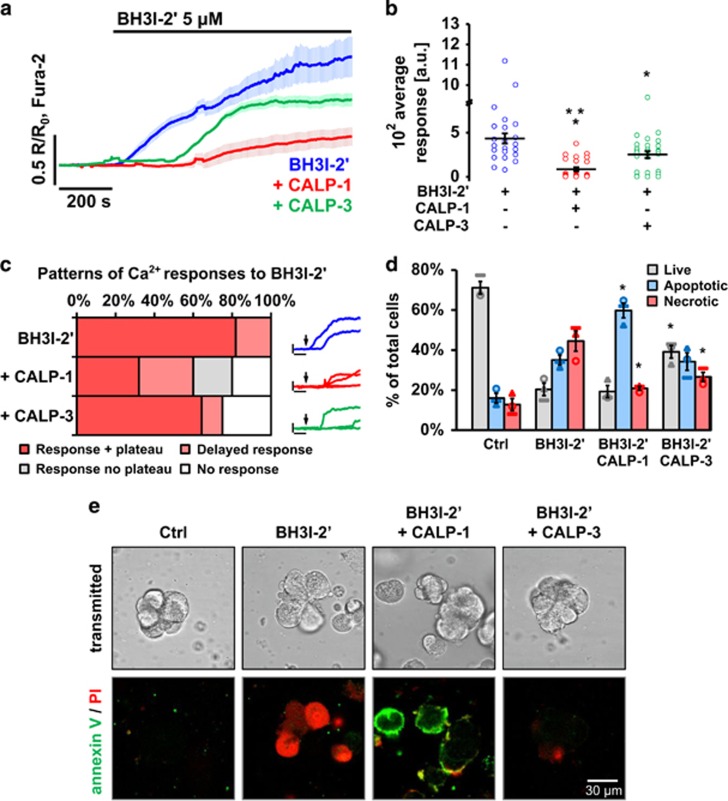
CALPs reduce Ca^2+^ responses and cell death induced by BH3I-2′. (**a**) Average Ca^2+^ responses (±S.E.M.) in PACs to 5 *μ*M BH3I-2′ (blue, *n*=22) and to BH3I-2′ in the presence of 100 *μ*M CALP-1 (red, *n*=25) or 100 *μ*M CALP-3 (green, *n*=28). (**b**) The responses shown in **a** were quantitatively analysed by comparing the average areas under traces recorded between 200 and 1000 s: control (blue, *n*=22, 434.1±57.6 a.u.), CALP-1 (red, *n*=25, 85.9±22.3 a.u.) and CALP-3 (green, *n*=28, 252.3±39.2 a.u.). (**c**) Distribution of different types of Ca^2+^ responses (averaged in **a**) induced by 5 *μ*M BH3I-2′ in the presence/absence of 100 *μ*M CALP-1 or CALP-3. Insets show sample traces of different response patterns; colours and scale values (*x* axis: 200 s; *y* axis: 0.5 *R/R*_0_, Fura-2) correspond with those shown in **a**. The mimetic alone predominantly induced a slow rise in [Ca^2+^]_i_, which started soon after the application. Eventually a steady elevated plateau was attained in the vast majority of cases (82%). In a small proportion of cells, the initial [Ca^2+^]_i_ rise was delayed (18%). In the presence of CALP-1, similar large responses reaching an elevated [Ca^2+^]_i_ plateau were recorded in only 32% of cells and 28% of the responses were delayed. Importantly, 20% of the cells responded only with a single cytosolic Ca^2+^ transient with no plateau formation, and the remaining 20% of the cells did not respond within the time course of the experiment. In the presence of CALP-3, 64% of the cells showed a typical [Ca^2+^]_i_ elevation in response to BH3I-2′, in 11% of cells the responses were delayed, and the remaining 25% did not respond at all. (**d**) Cell death induced by 30 min incubation with 5 *μ*M BH3I-2′ in PACs after 15 min pretreatment with 100 *μ*M CALP-1 or CALP-3. Grey bars represent live cells, blue – apoptotic cells and red – necrotic. *N*=3 for all the groups; individual values are shown with different markers (O,▴,▭). (**e**) Sample images show typical annexin V (green) and propidium iodide (red) staining of PACs used in the experiment shown in **d**

Excessive cytosolic Ca^2+^ signals can trigger necrosis, associated with release of intracellular content followed by inflammation.^34^ Necrosis is especially dangerous for the pancreas, where released activated digestive enzymes cause a serious threat for the integrity of the tissue.^35, 36^ Therefore apoptosis, executed by cells in a controlled manner, is a more favourable pathway for killing cells. Here, CALPs were applied in an attempt to reduce elevations in [Ca^2+^]_i_ and necrosis caused by BH3 mimetics. Preincubation of PACs either with 100 *μ*M CALP-1 or CALP-3 led to a decrease in the average amplitude of the Ca^2+^ signals occurring in response to BH3I-2′ (*P*<0.001 and *P*<0.05, respectively) compared to the control (average traces: Figure 5a; quantitative analysis: Figure 5b). This was due to a shift in the pattern of cytosolic Ca^2+^ signals elicited by 5 *μ*M BH3I-2′ (Figure 5c). The consequences of the CALP-mediated reductions in the cytosolic Ca^2+^ signal generation are reflected by the cell death pattern (Figures 5d and e). In the untreated control the majority of cells were alive (low levels of apoptosis and necrosis are due to enzymatic digestion of the tissue). Incubation with 5 *μ*M BH3I-2′ resulted in a 19.1±4.9% increase in apoptosis and a 31.7±8.1% increase in necrosis over the control levels (similar as in Figure 2f). In the presence of CALP-1, necrosis was significantly decreased (*P*<0.05), whereas apoptosis markedly increased (*P*<0.05) compared to BH3I-2′ alone. In contrast, CALP-3 did not affect apoptosis, but caused a reduction in necrotic cells (*P*<0.05) accompanied by an increase in the proportion of live cells (*P*<0.05).

### CALPs reduce excessive cytosolic Ca^2+^ responses and necrosis elicited by gossypol

**Figure 6:**
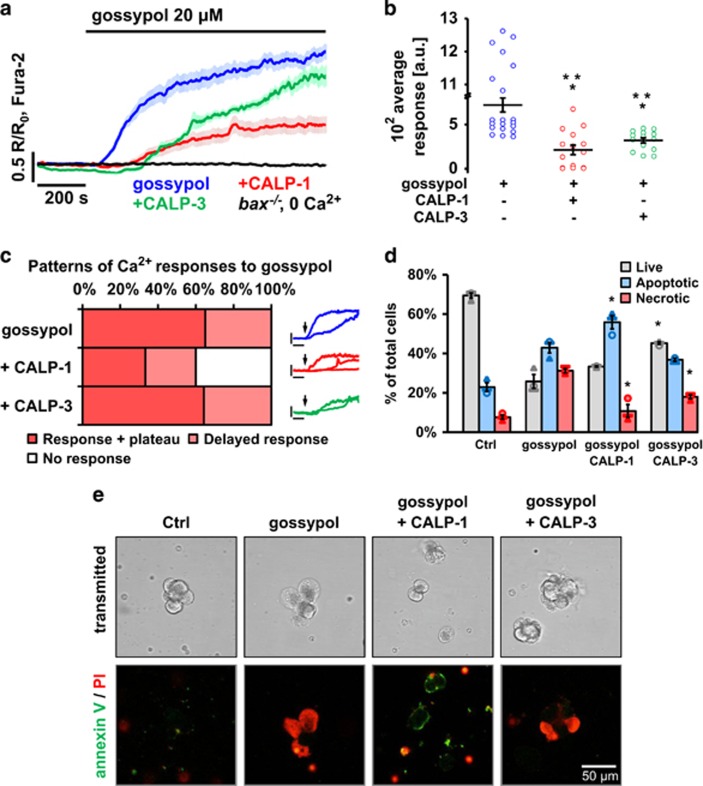
CALPs reduce Ca^2+^ responses and cell death induced by gossypol. (**a**) Average Ca^2+^ responses (±S.E.M.) in PACs to 20 *μ*M gossypol (blue, *n*=20) and to gossypol in the presence of 100 *μ*M CALP-1 (red, *n*=15) or CALP-3 (green, *n*=14); black trace shows lack of responses to 20 *μ*M gossypol in *bax*^−/−^ PACs in the absence of extracellular Ca^2+^ (*n*=3). (**b**) The responses shown in (**a**) were quantitatively analysed by comparing the average areas under traces recorded between 200 and 1000 s: control (blue, *n*=20, 721.1±79.4 a.u.), CALP-1 (red, *n*=15, 213.4±55.1 a.u.) and CALP-3 (green, *n*=14, 320.6±29.6 a.u.). (**c**) Distribution of different types of Ca^2+^ responses (averaged in **a**) induced in PSCs by 20 *μ*M gossypol in the presence/absence of 100 *μ*M CALP-1 or CALP-3. Insets show sample traces of different response patterns; colours and scale values (*x* axis: 200 s; *y* axis: 0.5 *R/R*_0_, Fura-2) correspond with those shown in **a**. 65% of the cells responded immediately, whereas responses in 35% of cells were delayed. CALP-1 decreased the proportion of cells that responded to gossypol either immediately (33%) or with a delay (27%) and as many as 40% of cells did not show any [Ca^2+^]_i_ elevation. In contrast, CALP-3 did not affect the pattern of the responses to gossypol – all cells developed either immediate (64%) or delayed (36%) elevations in [Ca^2+^]_i_, but of a lower amplitude than in cells treated with gossypol alone (shown in **a**). (**d**) Apoptosis and necrosis induced by 30 min incubation with 5 *μ*M gossypol in PACs after 15 min pretreatment with 100 *μ*M CALP-1 or CALP-3. Grey bars represent live cells, blue – apoptotic cells and red – necrotic. *N*=3 for all the groups; individual values are shown with different markers (O,▴,▭). (**e**) Sample images show typical annexin V (green) and propidium iodide (red) staining of PACs used in the experiment shown in **d**

It has been reported that gossypol is also capable of mobilising intracellular Ca^2+^.^37, 38^ Similarly to BH3I-2′ and HA14-1, in the absence of extracellular Ca^2+^, *bax*^−/−^ cells did not respond to gossypol (Figure 6a, black trace). In WT cells in the presence of external Ca^2+^, 20 *μ*M gossypol alone caused an increase in [Ca^2+^]_i_ reaching a sustained elevated plateau (Figure 6a). Preincubation either with 100 *μ*M CALP-1 or with CALP-3 led to a decrease in the magnitude of the Ca^2+^ responses to gossypol (Figures 6a and b; *P*<0.001); CALP-1 markedly decreased the proportion of cells that responded at all (Figure 6c). The effects of CALP-1 and CALP-3 on gossypol-induced cell death (Figures 6d and e) were very similar to those for BH3I-2′ (Figure 5d). In the presence of CALP-1, necrosis was markedly decreased (*P*<0.05) and the proportion of apoptotic cells was increased (*P*<0.05). CALP-3 reduced gossypol-induced necrosis (*P*<0.05) and increased the fraction of live cells (*P*<0.05).

### CALPs do not markedly affect physiological Ca^2+^ signals in acinar cells

**Figure 7:**
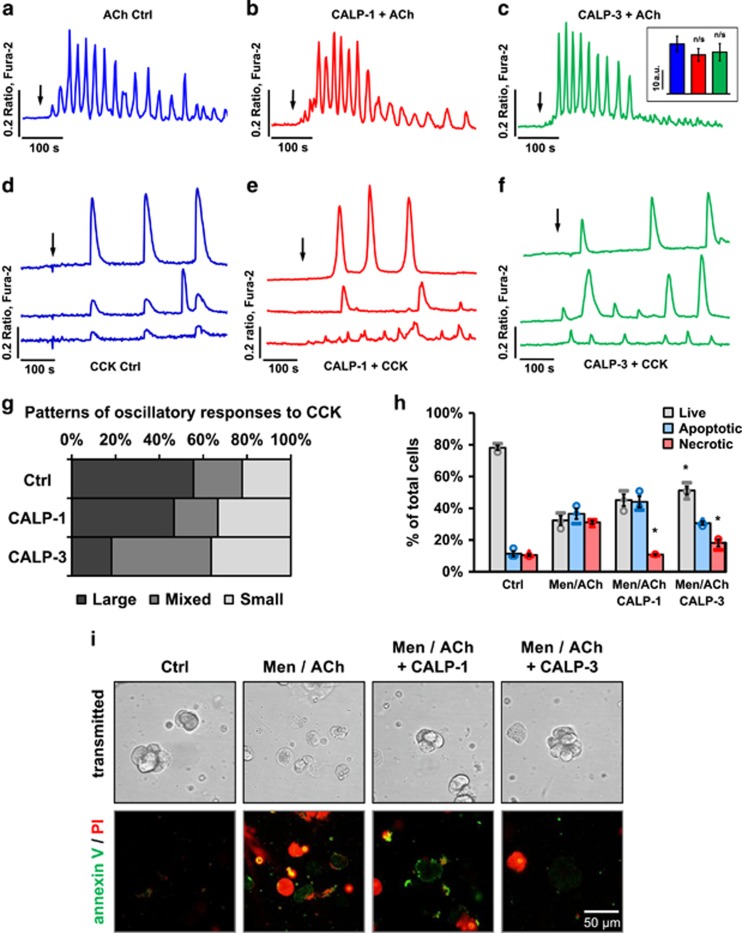
CALPs do not inhibit physiological Ca^2+^ responses in PACs. (**a**) 50 nM ACh induces Ca^2+^ oscillations in PACs (representative trace, *n*=15). (**b**) 50 nM ACh induces Ca^2+^ oscillations in PACs in the presence of 100 *μ*M CALP-1 (representative trace, *n*=12). CALP-1 was present 200 s before the addition of ACh. (**c**) 50 nM ACh induces Ca^2+^ oscillations in PACs in the presence of 100 *μ*M CALP-3 (representative trace, *n*=10). CALP-3 was present 200 s before the addition of ACh. Inset shows average areas under traces (±S.E.M.) calculated for 600 s after response induction by ACh (blue, 25.8±4.0 a.u.), ACh in the presence of CALP-1 (red, 20.2±3.3 a.u.) and ACh in the presence of CALP-3 (green, 21.6±4.5 a.u.). (**d**) Typical large Ca^2+^ transients (upper trace), mixed responses (middle trace) and small oscillations (lower trace) induced by 5 pM CCK in PACs (representative traces, *n*=9). Black arrow indicates addition of CCK. (**e**) Typical large Ca^2+^ transients (upper trace), mixed responses (middle trace) and small oscillations (lower trace) induced by 5 pM CCK in PACs in the presence of 100 *μ*M CALP-1 (representative traces, *n*=15). CALP-1 was present 200 s before the addition of CCK. (**f**) Typical large Ca^2+^ transients (upper trace), mixed responses (middle trace) and small oscillations (lower trace) induced by 5 pM CCK in PACs in the presence of 100 *μ*M CALP-3 (representative traces, *n*=11). CALP-3 was present 200 s before the the addition of CCK. (**g**) Distribution of different types of Ca^2+^ responses (representative traces shown in **d**–**f**) induced by 5 pM CCK in the presence/absence of 100 *μ*M CALP-1 or 100 *μ*M CALP-3. The majority of the control responses (56%) were large transients, 22% were small oscillations and the remaining 22% consisted of both large and small Ca^2+^ spikes. Preincubation with 100 *μ*M CALP-1 led to a slight decrease in the proportion of cells that responded with large Ca^2+^ oscillations (47%) accompanied by an increase in small responses (33%). CALP-3 reduced large transients even further (18%) resulting in higher proportions of cells responding with both large and small (45%), or small oscillations only (36%). (**h**) Apoptosis and necrosis induced by 30 min incubation with 5 *μ*M menadione (Men) and 100 nM ACh in PACs after 15 min pretreatment with 100 *μ*M CALP-1 or CALP-3. Grey bars represent live cells, blue – apoptotic cells and red – necrotic. *N*=3 for all the groups; individual values are shown with different markers (O,▴,▭). **(i)** Sample images show typical annexin V (green) and propidium iodide (red) staining of PACs used in the experiment shown in **h**

The ability of CALPs to reduce excessive pathological Ca^2+^ signal generation is potentially very useful but given the crucial role of Ca^2+^ in the regulation of a wide variety of intracellular processes, it was important to test whether CALPs also affect physiological Ca^2+^ signalling. Nanomolar doses of ACh trigger enzyme secretion in PACs, an event controlled by Ca^2+^ signals.^24^ Here, 50 nM ACh induced repetitive [Ca^2+^]_i_ oscillations (Figure 7a). Preincubation with either 100 *μ*M CALP-1 (Figure 7b) or CALP-3 (Figure 7c) did not affect the ACh-elicited signals. The average areas of the responses to ACh in the presence and absence of CALPs were not found significantly different (Figure 7c, inset). Another secretagogue of the exocrine pancreas,^39^ CCK (5 pM), induced oscillatory Ca^2+^ responses in acinar cells (Figure 7d). The presence of either CALP-1 (Figure 7e) or CALP-3 (Figure 7f) did not block the CCK-elicited responses, but the overall pattern was slightly changed (Figure 7g).

### The effects of CALPs on cell death are not limited to BH3-mediated necrosis

As the primary mechanism by which CALPs exert their effects is the pre-activation of EF hand Ca^2+^-binding sites on a wide variety of intracellular targets^29^ and inhibition of Ca^2+^ entry (Figures 4a–d), it is unlikely that CALP-mediated reduction in excessive Ca^2+^ signals and necrosis is limited only to the effects induced by BH3 mimetics. Menadione was previously shown to induce Ca^2+^-dependent cell death in PACs.^40^ Here, 5 *μ*M menadione was combined with a high physiological concentration of ACh (100 nM) in order to induce Ca^2+^-dependent cell death of similar proportions of apoptosis and necrosis (25.1±5.0% and 20.7±1.7%, respectively; Figure 7h; sample images in Figure 7i) to those caused by BH3I-2′ (Figure 5d) or gossypol (Figure 6d). In this protocol 100 *μ*M CALP-1 decreased cell necrosis to control levels (*P*<0.05) while increasing slightly the proportions of both live and apoptotic cells; whereas 100 *μ*M CALP-3 substantially reduced necrosis (*P*<0.05), leading to an increase in the number of live cells (*P*<0.05). The apoptotic fraction was essentially unaffected by CALP-3.

## Discussion

It is now commonly accepted that Bcl-2 proteins are distributed in different cell compartments, not only at the outer mitochondrial membrane, but also in the cytosol, at the nuclear envelope and the ER.^41, 42, 43^ An increasing number of reports indicates that Bcl-2 itself may either directly or indirectly modulate intracellular Ca^2+^ fluxes such as: (1) Ca^2+^ release from the ER by binding to IP_3_Rs or RyRs;^44, 45, 46^ (2) Ca^2+^ reuptake into the ER by SERCA;^47^ (3) cellular Ca^2+^ extrusion by PMCA (plasma membrane Ca^2+^ ATPase);^48^ (4) and mitochondrial Ca^2+^ load.^49^

We have previously demonstrated that the BH3 mimetics BH3I-2′ and HA14-1 induce Ca^2+^ release from the intracellular stores in mouse PACs and in the rat pancreatic cancer cell line AR42J.^16^ This Ca^2+^ release was not completely blocked by inhibition of IP_3_Rs and RyRs indicating involvement of other Ca^2+^ channels. We have also shown that BH3I-2′ and HA14-1 displace Bax from Bcl-2 and Bcl-xL.^16^ Here we provide new insights into the mechanism of this phenomenon by demonstrating that loss of Bax, but not Bak or Bcl-2, substantially inhibited Ca^2+^ responses to BH3I-2′ (Figure 1a), HA14-1 (Figure 1b) and gossypol (Figure 6a) in the absence of extracellular Ca^2+^. HA14-1 and gossypol were suggested to have off-target effects,^50, 51^ and recently a novel Bcl-2 inhibitor, ABT-199, was found not to affect intracellular Ca^2+^ signalling.^52^ In our experiments not only the responses to HA14-1 and BH3I-2′ were markedly inhibited in *bax*^−/−^ PACs (Figures 1a and b) but also BH3I-2′-elicited apoptosis was completely abolished (Figure 2f), indicating that these effects were dependent on Bax. Low levels of necrosis in *bax*^−/−^ cells may indeed suggest a Bax-independent killing component in the mechanism of BH3I-2′ action. Importantly, BH3I-2′-induced cell death in PACs appears to be mediated by Ca^2+^ as it was blocked by chelation of intracellular Ca^2+^ with BAPTA (Figure 2f).

**Figure 8:**
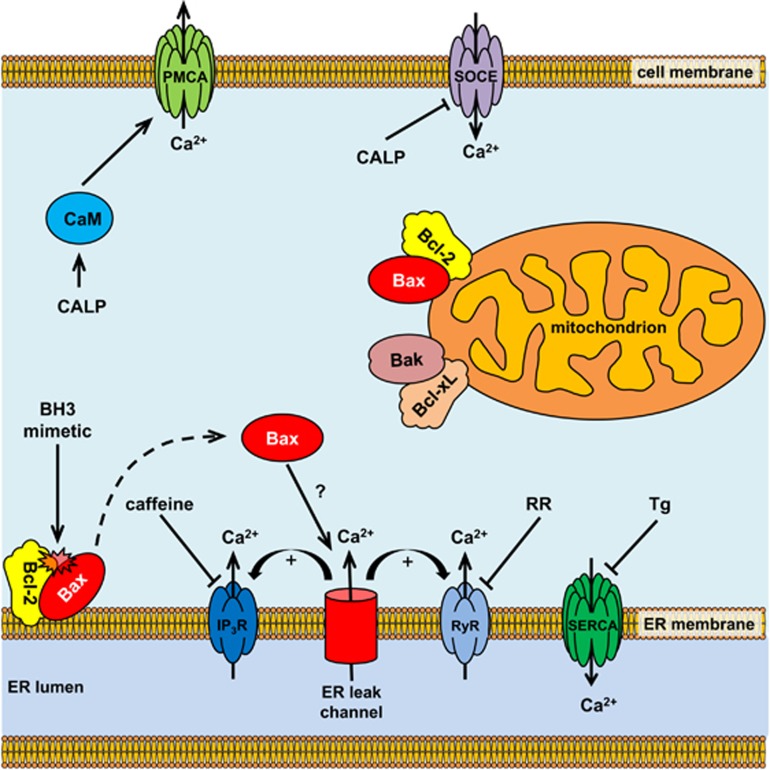
Schematic illustration of proposed effects of BH3 mimetics and CALPs on intracellular Ca^2+^ homoeostasis. BH3 mimetics (such as BH3I-2′ or gossypol) disrupt the interaction between anti-apoptotic Bcl-2 and pro-apoptotic Bax. Unbound Bax may either form channels in the ER membranes or interact with the ER leak channel triggering Ca^2+^ release from the ER stores. Ca^2+^ release is amplified by IP_3_Rs and RyRs. These receptors can be inhibited by caffeine and ruthenium red (RR), respectively. CALPs affect multiple targets via interaction with their EF hand motifs, for example, inhibit SOCE and activate calmodulin (CaM)

Our results appear to link Bax with the process of passive Ca^2+^ leak from the ER. The leak, unmasked by thapsigargin, developed more slowly in *bax*^−/−^ cells compared to WT cells (Figures 3c and e). Currently there is an on-going dispute about the nature of the channels mediating this process. As Bax can localise to the ER membranes^53^ and given that its structure resembles that of pore-forming bacterial toxins,^54, 55^ Bax itself might be able to form a channel permeable to ions and thus contribute to the passive Ca^2+^ leak. Two out of seven *α*-helices of Bax were suggested to function as pore-forming domains, but possibly more than one monomer of Bax would need to form a functional ion-permeable pore.^56, 57^ Alternatively, as opposed to acting as a channel itself, Bax could directly or indirectly regulate the ER Ca^2+^ leak mediated by other channels. Consequently, it could be speculated that application of a BH3 mimetic increases the unbound fraction of Bax, which activates Ca^2+^ channels in the ER, thus potentiating the leak (Figure 8). The leak may be further amplified by IP_3_Rs and RyRs.

The reduced cytosolic Ca^2+^ release in response to thapsigargin treatment in *bax*^−/−^ cells could, in principle, be explained by a diminished resting ER Ca^2+^ content. However, as ACh-induced oscillations were essentially unaffected in *bax*^−/−^ cells (Figures 3h–j), this may indicate that the [Ca^2+^]_ER_ has not been substantially affected by loss of Bax. Previous experiments demonstrated that ACh-evoked short-lasting Ca^2+^ spikes in PACs are very sensitive to even minor reductions in [Ca^2+^]_ER_ and cease after a relatively modest decrease in [Ca^2+^]_ER_.^58^ This could shed new light on a seeming discrepancy found in the previous studies, whereby both knockdown^59, 60^ and overexpression^61, 62^ of Bax led to a reduction in Ca^2+^ responses induced by various agonists or inhibition of SERCA. It is likely that upon loss of Bax, some of the leak channels are inactive, and those that remain active may be sensitive to changes in [Ca^2+^]_ER_ and, for example, close early in the release period thereby limiting Ca^2+^ release from the ER. In contrast, overexpression of Bax may lead to an increased basal leak, which results in a decrease in resting [Ca^2+^]_ER_ and thus dampens Ca^2+^ responses. Finally, under normal conditions, Bax is sequestrated by anti-apoptotic Bcl-2 family proteins. But any shifts in the balance between anti- and pro-apoptotic Bcl-2 members may promote mechanisms that either favour pro-survival Ca^2+^ transients or larger pro-apoptotic Ca^2+^ signals.^63^

Further, our results show that the BH3 mimetics, BH3I-2′ and gossypol not only induce apoptosis in PACs but also cause substantial levels of necrosis (Figures 5d and 6d). Cell death in PACs was a downstream effect of large cytosolic Ca^2+^ signals triggered by application of BH3 mimetics, as (1) Ca^2+^ chelation by BAPTA completely abolished both apoptosis and necrosis induced by BH3I-2′ and (2) reduction of Ca^2+^ signals elicited by CALP-1 or CALP-3 (Figures 5a and 6a) led to a significant decrease in BH3I-2′- as well as gossypol-induced necrosis (Figures 5d and 6d). CALP-1 was shown to be particularly promising, as it effectively shifted the cell death mode from necrosis to apoptosis. In contrast, CALP-3 substantially reduced BH3 mimetic-induced necrosis while increasing the fraction of live cells. Both CALPs were capable of inhibiting Ca^2+^ entry (Figures 4a–d), even though they differ in structure. CALP-1 has been designed to interact with EF hand Ca^2+^-binding sites of troponin C, whereas CALP-3 is complementary to the EF hand motif of calmodulin.^32^ It is therefore likely that the two CALPs might preferentially interact with different intracellular targets, for example, Ca^2+^ channels regulated by Ca^2+^ *versus* calmodulin, abundant in PACs.^64^ This could explain different effects on BH3 mimetic-induced cell death. Previously we demonstrated that CALP-3 efficiently inhibited Ca^2+^ responses induced by ethanol in PACs and we attributed this effect to activation of calmodulin.^65^ The exact assessment of binding affinities of CALPs to intracellular targets exceeds the scope of this work. It is clear, however, that the effects of CALPs on cell death are not limited to BH3 mimetics, as necrosis triggered by ACh and menadione was also inhibited in the presence of CALPs (Figure 7h).

Considering the vast spectrum of targets affected by CALPs, it was important to test whether application of these compounds would affect not only pathological Ca^2+^ elevations but also physiological Ca^2+^ signals. In PACs, nanomolar doses of ACh and picomolar concentrations of CCK are known to trigger Ca^2+^ oscillations, which regulate pancreatic enzyme secretion.^24^ In our experiments neither CALP-1 nor CALP-3 markedly affected responses to ACh (Figures 7a–c), whereas in the presence of CALP-3, CCK-elicited Ca^2+^ oscillations were slightly reduced but not completely inhibited (Figures 7d–g). The previously demonstrated inhibition of Ca^2+^ influx into PACs by the CRAC channel inhibitor GSK-7975A also affected only pathological Ca^2+^ elevations but not the oscillations induced by physiological concentrations of ACh or CCK.^66^

Although it can be triggered by sustained elevations of [Ca^2+^]_i_, necrosis generally lacks intracellular regulatory mechanisms. This severely limits possible therapeutic approaches in diseases where necrosis plays a major role, such as acute pancreatitis. Inhibition of SOCE has already been demonstrated to decrease cell death *in vitro*^66^ and *in vivo*.^67^ This study provides another proof-of-principle demonstration that even non-specific attenuation of intracellular Ca^2+^ fluxes can dampen pathophysiological Ca^2+^ responses and thus result in necrosis inhibition (CALP-3) or a significant shift in cell death towards apoptosis (CALP-1).

Our results indicate that application of CALPs, especially CALP-1, could improve the outcome of BH3 mimetic-based therapies. Although the pharmacokinetic properties of CALPs have not yet been studied in detail, it is likely that CALPs share characteristics of other short peptides, such as limited cell permeability and metabolic instability.^68^ However, development of synthetic non-peptide compounds activating EF hand Ca^2+^-binding motifs could potentially overcome these limitations, providing a useful pharmacological switch of cell death mode, particularly in cancer therapies.

## Materials and Methods

### Reagents

The main reagents for cell isolation and imaging include: Fluo-4 AM, Fura-2 AM, Fluo-5N AM and BAPTA AM (ThermoFisher Scientific, Paisley, UK); collagenase (Worthington Biochemical Corporation, Lakewood, NJ, USA); inorganic salts (Sigma-Aldrich, Gillingham, UK). Other reagents: HA14-1 (Alexis Biochemicals, San Diego, CA, USA); BH3I-2′, gossypol (Santa Cruz Biotechnology, Dallas, TX, USA); caffeine and thapsigargin (Calbiochem, Nottingham, UK); CALP-1, CALP-3, ruthenium red and CDN1163 (Tocris Bioscience, Bristol, UK). NaHEPES buffer was prepared as follows (mM): NaCl 140, KCl 4.7, HEPES 10, MgCl_2_ 1, glucose 10; pH 7.2. KHEPES (intracellular solution) consisted of (mM): KCl 130, NaCl 18, MgCl_2_ 1, HEPES 10, ATP 3, EGTA 0.1, CaCl_2_ 0.05; pH 7.2.

### Animals

All procedures involving animals were performed in accordance with the UK Home Office regulations. C57BL/6J mice (male, 6–8 weeks old, 23±3 g weight) were supplied by Charles River Laboratories (Margate, UK); transgenic mice *bcl-2*^−/−^ (B6;129S2-BCL-2^tm1Sjk^/J), *bax*^−/−^ (B6.129X1-Bax^tm1Sjk^/J) were obtained from The Jackson Laboratories (Bar Harbor, ME, USA); *bak*^−/−^ (B6.129-Bak1^tm1Thsn^/J) colony was a gift of Professor David Mark Pritchard (University of Liverpool); *bax*^−/−^ strain extensively crossed into the CD1 background was also obtained from Professor Alun Davies (Cardiff University).^69^ Transgenic mice were bred in house either from null (*bak*^−/−^) or heterozygous (*bcl-2*^−/−^, *bax*^−/−^) parents; the genotype of each mouse was confirmed by PCR reaction with primers suggested by the supplier. All animals were housed in the institutional animal unit, maintained on a 12 h light cycle on a standard rodent chow diet with free access to water. The mice were killed according to Schedule 1 of Animals (Scientific Procedures) Act 1986, dissected and the pancreatic tissue was removed for further experimental procedures.

### Isolation of pancreatic acinar cells

PAC isolation and most of the experimental work was carried out in NaHEPES buffer. Unless otherwise stated, NaHEPES was supplemented with 1 mM Ca^2+^. Freshly isolated pancreas was washed twice in NaHEPES, injected with collagenase (200 μ/ml, in NaHEPES) and subsequently incubated at 37 °C for 15 min in the collagenase solution to allow digestion of the tissue. After incubation, the pancreas was broken down by pipetting, suspended in NaHEPES, spun (1 min, 0.2 × *g*), resuspended in NaHEPES and spun again. Finally, isolated PACs were suspended in NaHEPES and loaded with a Ca^2+^ sensitive dye as described below.

### Cytosolic Ca^2+^ measurements

Isolated PACs were loaded at room temperature with one of the Ca^2+^ indicators: 5 *μ*M Fluo-4 AM for 30 min or 10 *μ*M Fura-2 AM for 1 h. After the incubation the cells were resuspended in fresh NaHEPES and used for experiments at room temperature in a flow chamber perfused with NaHEPES-based extracellular solution. Experiments with Fluo-4 AM were performed using the Leica confocal microscope TCS SPE (Leica Microsystems, Milton Keynes, UK): × 63 oil objective, excitation 488 nm, emission 500–600 nm. Static images were taken at 512 × 512 pixel resolution and series of images were recorded at 256 × 256 pixel resolution, two consecutive frames were averaged. Fluorescence signals were plotted as *F/F*_0_, where *F*_0_ was an averaged signal from the first ten baseline images. Experiments with Fura-2 AM were performed using the Nikon Diaphot 200 imaging system (Nikon, Kingston, UK): excitation at 365 and 385 nm, emission at 510 nm. The signals were plotted as 365/385 nm ratio or the ratio normalized to baseline values (*R/R_0_*).

### ER Ca^2+^ measurements

PACs were loaded with 5 *μ*M Fluo-5N AM in NaHEPES for 45 min at 37 °C. After the incubation, the cells were resuspended in fresh NaHEPES and used for experiments at room temperature in a flow chamber perfused with KHEPES-based intracellular solution. The cells were permeabilised with two-photon laser beam (720–750 nm),^70^ which was applied at a small area of the plasma membrane of a PAC. The experiments were performed using the Leica two-photon confocal microscope TCS SP5 with the same settings as for Fluo-4 AM (see above).

### Cell death assay

Cell death assay was performed using Annexin V-FITC Apoptosis Detection Kit (Sigma-Aldrich) according to a modified manufacturer′s protocol. PACs were isolated as described above and divided equally into four experimental groups. Two samples were pretreated with 100 *μ*M CALP-1 or 100 *μ*M CALP-3 for 15 min at room temperature; the remaining two were incubated without pretreatment. Then cell death was induced for 30 min in both samples containing CALPs and in one untreated sample. Depending on the experiment, either one of BH3 mimetics was used (5 *μ*M BH3I-2′ or 5 *μ*M gossypol) or 5 *μ*M menadione together with 100 nM ACh. Analogical experiments were performed with 15 min pretreatment with BAPTA instead of CALPs. The control samples were left untreated. Fifteen  minutes before the end of the incubation, annexin V-FITC and propidium iodide were added to all samples. The cells were visualised with the Leica confocal microscope TCS SPE. Annexin V-FITC specifically stained apoptotic cells (excitation: 488 nm, emission: 510–570 nm), whereas propidium iodide was used for detection of necrotic cells (excitation: 535 nm, emission: 585–705 nm). Multiple pictures (20–35) per treatment group were taken; live, apoptotic and necrotic cells were counted in each treatment group.

### Statistical analysis

For quantitative analysis of Ca^2+^ responses, areas under individual traces were calculated according to the formula: 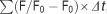, where *F* is the recorded fluorescence (or ratio for Fura-2), *F*_0_ is the baseline fluorescence (or ratio) and Δ*t* – time interval. Obtained values were then averaged and presented as bar charts with S.E.M. The Student′s *t*-test was applied for statistical comparison. The significance threshold was set at 0.05 and the range was indicated by asterisks (*P<0.05, **P<0.01, ***P<0.001). Where applicable, *N* indicates the number of individual experiments, whereas *n* – individual cells.

For cell death assays, three independent experiments were performed for each treatment group; average values and S.E.M. were calculated and the results presented as bar charts. Statistical analysis was performed using the non-parametric Mann–Whitney *U-*test with the significance threshold set at 0.05.
